# Age-related increase in the expression of 11β-hydroxysteroid dehydrogenase type 1 in the hippocampus of male rhesus macaques

**DOI:** 10.3389/fnagi.2024.1328543

**Published:** 2024-03-15

**Authors:** Alejandro Lomniczi, Selva L. Luna, Rita Cervera-Juanes, Maria-Luisa Appleman, Steven G. Kohama, Henryk F. Urbanski

**Affiliations:** ^1^Department of Physiology and Biophysics, Dalhousie University, Halifax, NS, Canada; ^2^Escuela de Química y Farmacia, Facultad de Farmacia, Universidad de Valparaíso, Valparaíso, Chile; ^3^Department of Physiology and Pharmacology, Atrium Health Wake Forest Baptist Medical Center, Winston-Salem, NC, United States; ^4^Division of Neuroscience, Oregon National Primate Research Center, Beaverton, OR, United States; ^5^Division of Reproductive and Developmental Sciences, Oregon National Primate Research Center, Beaverton, OR, United States; ^6^Department of Behavioral Neuroscience, Oregon Health and Science University, Portland, OR, United States

**Keywords:** cortisol, cortisone, *HSD11B1*, *NR3C1*, *NR3C2*, RNA-Seq

## Abstract

**Introduction:**

The hippocampus is especially susceptible to age-associated neuronal pathologies, and there is concern that the age-associated rise in cortisol secretion from the adrenal gland may contribute to their etiology. Furthermore, because 11β-hydroxysteroid dehydrogenase type 1 (HSD11B1) catalyzes the reduction of cortisone to the active hormone cortisol, it is plausible that an increase in the expression of this enzyme enhances the deleterious impact of cortisol in the hippocampus and contributes to the neuronal pathologies that underlie cognitive decline in the elderly.

**Methods:**

Rhesus macaques were used as a translational animal model of human aging, to examine age-related changes in gene and protein expressions of (*HSD11B1*/HSD11B1) in the hippocampus, a region of the brain that plays a crucial role in learning and memory.

**Results:**

Older animals showed significantly (*p* < 0.01) higher base-line cortisol levels in the circulation. In addition, they showed significantly (*p* < 0.05) higher hippocampal expression of *HSD11B1* but not *NR3C1* and *NR3C2* (i.e., two receptor-encoding genes through which cortisol exerts its physiological actions). A similar age-related significant (*p* < 0.05) increase in the expression of the HSD11B1 was revealed at the protein level by western blot analysis.

**Discussion:**

The data suggest that an age-related increase in the expression of hippocampal *HSD11B1* is likely to raise cortisol concentrations in this cognitive brain area, and thereby contribute to the etiology of neuropathologies that ultimately lead to neuronal loss and dementia. Targeting this enzyme pharmacologically may help to reduce the negative impact of elevated cortisol concentrations within glucocorticoid-sensitive brain areas and thereby afford neuronal protection.

## 1 Introduction

The hippocampus (HC) is particularly vulnerable to several age-related neuro-pathologies, including Alzheimer’s disease (AD) and limbic-predominant age-related TDP-43 encephalopathy (LATE) (Stonebarger et al., 2021; Nilaver and Urbanski, 2023). Although the root causes of these pathologies are unclear, there is evidence that glucocorticoids, such as cortisol, may potentiate neurotoxicity in this brain area and thereby contribute to their etiology (Lupien et al., 1998; Hibberd et al., 2000; McEwen, 2002; Herbert et al., 2006). Possible ways in which this potentiation could be achieved include an increase in circulating cortisol levels, derived from the adrenal gland, and/or an increase in the densities of neuronal glucocorticoid receptors (GRs) and mineralocorticoid receptors (MRs). However, an additional possibility involves increased local synthesis or reduced clearance of active glucocorticoids from the brain, stemming from an age-related increase in the expression of 11 beta-hydroxysteroid dehydrogenase type 1, which is encoded by HSD11B1 (Chapman et al., 2013). This enzyme is responsible for the conversion of cortisone, a less neurotoxic steroid, to cortisol. Therefore, an increase in the expression of *HSD11B1* would potentiate the negative effects of chronically elevated cortisol concentrations in brain areas where it is expressed and where cortisol is known to act (Seckl et al., 2002; Holmes et al., 2003; Seckl, 2004; Holmes and Seckl, 2006). In support of this idea are several rodent studies showing that HSD11B1 can affect cortisol levels in the hippocampus and in turn impair cognitive function (Yau et al., 2006; MacLullich et al., 2012). On the other hand, very few studies have been performed in nonhuman primates to show if similar mechanisms are likely to operate in humans and contribute to age-associated brain pathologies in the elderly.

Like humans, rhesus macaques are long-lived primates and show similar brain organization and development. Furthermore, the brains of old rhesus macaques, like elderly humans, progressively show many of the same pathological hallmarks of Alzheimer’s disease, including Aβ plaques and phosphorylated Tau protein (Uno, 1993; Paspalas et al., 2018; Stonebarger et al., 2021; Appleman et al., 2024). Importantly, however, rhesus macaques can be maintained under tightly controlled environmental conditions, and with a minimal post-mortem interval for tissue collection. Consequently, they represent a valuable translational animal model for gaining insights into mechanisms that underlie human aging (Messaoudi et al., 2011; Stonebarger et al., 2021).

In support of a causal link between hippocampal *HSD11B1* and cognition, we previously showed that hippocampal expression of this gene was significantly greater in old female rhesus macaques that performed significantly worse on a delayed matching-to-sample memory task (Sorwell et al., 2017) that involves the hippocampus (Bachevalier et al., 1991; Buccafusco et al., 2002; Aizawa et al., 2009). We, therefore, hypothesized that the expression of *HSD11B1* should be significantly higher in older animals, both at the mRNA and at the protein level. To test this, we used RNA-seq to compare hippocampal expression of *HSD11B1* as well as cortisol-sensitive receptors *NR3C1* and *NR3C2,* in young and old male rhesus macaques. We also used western blot analysis to examine HSD11B1 protein expression in the same animals. The data suggest that an age-related increase in the expression of *HSD11B1* in vulnerable brain areas may predispose them to the neurotoxic influence of high cortisol levels.

## 2 Materials and methods

### 2.1 Animals

Postmortem brain tissue was collected from 6 young adult (8–15 years) and 5 old (23–28 years) male rhesus macaques (*Macaca mulatta*) that had previously been used in an Institutional Animal Care and Use Committee approved study (Urbanski, 2017). The animals had been cared for at the Oregon National Primate Research Center (ONPRC) in accordance with the National Research Council’s Guide for the Care and Use of Laboratory Animals. They were housed indoors in a temperature-controlled environment (24°C) under a 12L:12D photoperiod (lights on from 07:00 to 19:00 h). Daily meals at ~08:00 h and ~ 15:00 h (LabDiet High Protein Monkey Chow, St. Louis, MO, United States) were supplemented with fresh fruits or vegetables; fresh drinking water was available *ad libitum*.

### 2.2 Collection of serum

Prior to necropsy, serial blood samples were collected hourly from 4 of the young and 4 of the old males, for 25 h, using a remote unobtrusive blood sampling system (Urbanski, 2011; Urbanski et al., 2014). The serum was assayed for cortisol using a chemiluminescence-based automatic clinical platform (Roche Cobas e411, Roche Diagnostics, Indianapolis, IN, United States), and the *maximum* and *minimum* values were calculated as the average of 5 consecutive peak concentrations and 5 consecutive nadir concentrations, respectively.

### 2.3 Collection of brain tissue

A detailed necropsy protocol previously used in our laboratory was used to systematically collect brain tissues from all the animals (Cervera-Juanes et al., 2022); other body tissues were made available to other investigators for unrelated postmortem studies. The animals were sedated with ketamine (10 mg/kg), and administered pentobarbital, followed by exsanguination, as recommended by the 2013 Edition of the American Veterinary Medical Association Guidelines for the Euthanasia of Animals. The brains were flushed with 0.9% saline and the right hemisphere was dissected to isolate the different brain regions, including the hippocampus, which was approached medially; the mid-body hippocampal subregion was subsequently used in this study. All tissues were wrapped in aluminum foil and immediately frozen in liquid nitrogen, and then archived at −80°C.

### 2.4 Tissue preparation for extraction

100–200 mg of hippocampal tissue was pulverized, using a tissue pulverizer (Thomas Scientific, Swedesboro, NJ, United States), and mixed. To maintain molecular integrity, while reducing degradation, the samples were maintained on dry-ice throughout the procedure. They were then stored in microcentrifuge tubes at −80°C.

### 2.5 RNA-seq

#### 2.5.1 DNA/RNA isolation

Genomic DNA and RNA were extracted from the powdered hippocampal samples using the All-Prep DNA/RNA/miRNA Universal kit (Qiagen Sciences Inc., Germantown, MD, United States) following the manufacturer’s recommendations. Briefly, each brain region was pulverized and ~30 mg of tissue was used for DNA/RNA isolation. For stranded RNA-seq, cDNA libraries were prepared with the TruSeq stranded mRNA library prep Kit (cat# RS-122-2101, Illumina, San Diego, CA, United States). The resulting libraries were sequenced on a HiSeq 4000 (Genomics & Cell Characterization Core Facility, University of Oregon) using a paired-end run (2 × 150 bases). A minimum of 100 M reads was generated from each library.

Differential expression analysis: After verification of quality control of the sequences with FastQC (v. 0.1.1.2) (Andrews, 2010), sequences were aligned to the macaque Mmul_10 (INSDCA Assembly GCA_003339765.3) genome through the STAR alignment package (v. 2.7.3a) (Dobin et al., 2013). Read counts were obtained with STAR and based on the Ensembl.Mmul_10.100 genome annotation. edgeR (v. 3.28.0) (Robinson et al., 2010) from the Bioconductor package (Bioconductor, Cambridge, United Kingdom) were used for upper quartile normalization using the genes with a minimum of 0.3 CPM across samples. These thresholds translate into roughly 10 reads per minimum library size (10/minimum library size in millions) (Chen et al., 2016). Differential expression between the young and old groups was determined using edgeR’s Fisher’s exact test function, with the option of “tagwise” dispersion. The threshold for significance was unadjusted *p* = 0.05.

### 2.6 Western blot

#### 2.6.1 Tissue protein extraction

Approximately 50 mg of powdered hippocampal tissue was homogenized in 1 mL of ice-cold RIPA buffer (Thermo Fisher Scientific, Rockford, IL, United States) using a glass Dounce homogenizer. Buffers contained HALT Protease Inhibitor Cocktail and HALT Phosphatase Inhibitor Cocktail (Thermo Fisher Scientific). After a 10 min centrifugation at 16000 g, the supernatant was immediately flash frozen and stored at −80°C. Protein concentration was determined using the Micro BCA protein Assay Kit (Thermo Fisher Scientific), following the manufacturer’s instructions.

#### 2.6.2 Protein analysis

Protein lysates (100 μg) were subjected to non-reducing SDS-PAGE on 4–12% Bis-Tris polyacrylamide gels (Thermo Fisher Scientific), electro-transferred on polyvinylidene difluoride (PVDF) membranes (Thermo Fisher Scientific), blocked for 1 h at room temperature with a solution of 5% non-fat dry milk in Tris-Buffered Saline with Tween 20 (TBST) and probed overnight at 4°C in the presence of a rabbit polyclonal antibody directed against human HSD11B1 (Abcam AB39364) diluted 1:1000 in TBST, or GAPDH (Abcam, AB8245) at 1:10,000, a housekeeping protein, as a loading control. Molecular weights were estimated by running 7 μL of Seeblue Plus2 pre-stained protein ladder (LC5925, Thermo Fisher Scientific). For protein detection, we incubated the membranes in horseradish peroxidase-conjugated anti-rabbit secondary antibodies (Thermo Fisher Scientific) diluted 1:50,000 in TBST for 1 h at room temperature and West Dura chemiluminescence (Thermo Fisher Scientific). Chemiluminescence signal was detected and digitalized using a FluorChem System (Bio-Techne, Minneapolis, MN, United States).

#### 2.6.3 De-glycosylation of protein extracts

To test whether the different molecular weights of HSD11B1 are due to glycosylation, we performed a de-glycosylation experiment using the glycoprotein de-glycosylation kit from MilliporeSigma (Burlington, MA, United States, catalog #362280). Briefly, 200 μg protein extracts were incubated with reaction buffer and 1 μL each of N-Glycosidase F, α2-3,6,8,9-Neuraminidase, Endo-α-N-acetylgalactosaminidase, β1,4-galactosidase and 1 μL β-N-Acetylglucosaminidase and incubated for 3 h at 37°C. Protein detection was performed by western blot as described above.

### 2.7 Statistics

Group means were compared using unpaired Student’s *t*-test, with *p* < 0.05 being considered statistically significant. The relationship between *HSD11B1* mRNA and HSD11B1 protein concentrations was analyzed by Pearson’s correlation coefficient.

## 3 Results

In the present study we used a male rhesus macaque model to study the effect of normative aging on cortisol pathway dynamics, and focused on two cohorts of animals: A *young* group composed of 6 young adult (8–15 years) and an *old* group with 5 old (23–28 years) male rhesus macaques. To understand circulating cortisol dynamics, we collected hourly blood samples from 4 young and 4 old males for 25 h using a remote blood sampling system. While there was no statistically significant difference in average maximum cortisol levels, there was a significant (*p* < 0.01) increase in the average minimum circulating cortisol levels in the old group (Table 1). These results confirm that aging in rhesus macaques is associated with a dysregulation of the circadian control of cortisol, inducing an overall chronic increase of total cortisol exposure, which ultimately may prove to be neurotoxic in some brain areas.

**Table 1:** Serum concentration dynamics of cortisol in young and old male rhesus macaques.

Age group	Age (years)	Maximum (ng/mL)	Minimum (ng/mL)
Young (*n* = 4)	11.6 ± 1.2	206.1 ± 16.3	46.8 ± 6.0
Old (*n* = 4)	26.1 ± 0.8^***^	230.1 ± 19.2	77.4 ± 4.5^**^

Maximum = peak cortisol concentration, occurring during the early morning (mean ± SEM). Minimum = nadir cortisol concentration, occurring during the late evening (mean ± SEM). ^**^p < 0.01 and ^***^p < 0.001 (Student’s *t*-test).

**Figure 1:**
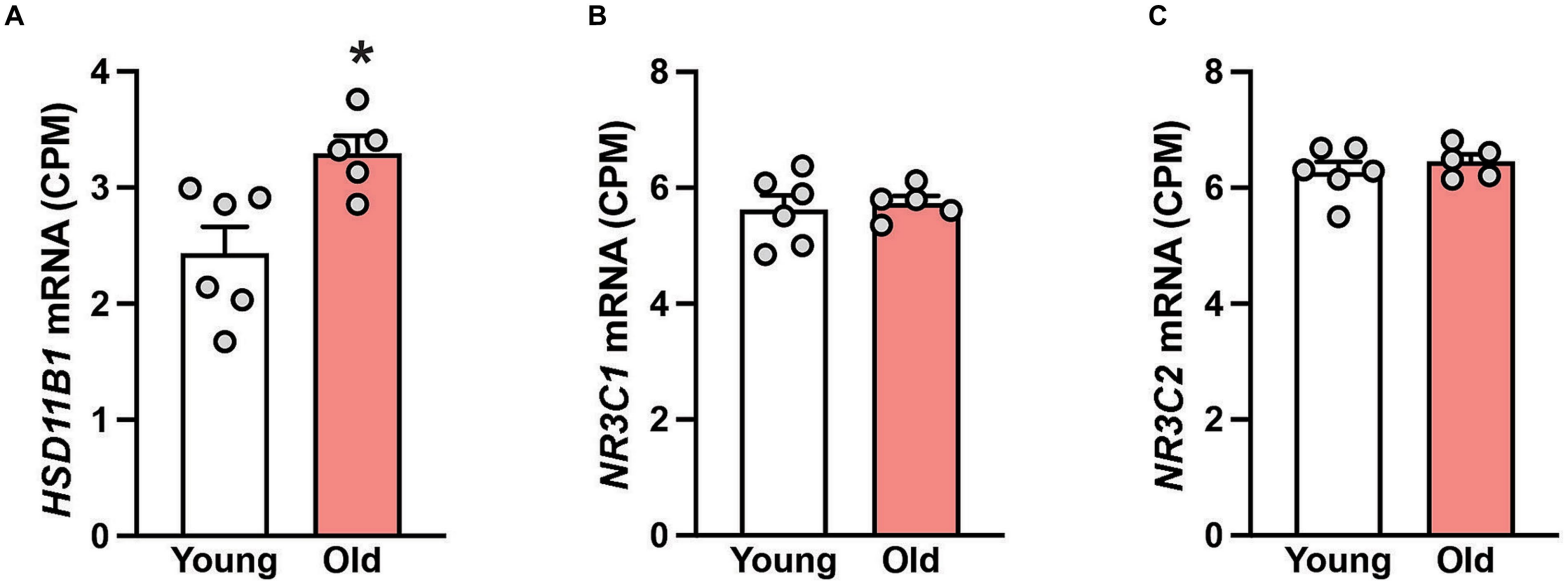
Gene expression in the hippocampus of 6 young and 5 old male rhesus macaques. Group means (+SEMs) are depicted for *HSD11B1* **(A)**, the glucocorticoid receptor *NR3C1* **(B)** and the mineralocorticoid receptor *NR3C2* **(C)**. ^*^p < 0.05 (Student’s *t*-test). The higher expression of *HSD11B1* in the old animals implies enhanced conversion of cortisone to cortisol. The consistent expression of *NR3C1* and *NR3C2* across age suggests that hippocampal neurons retain their sensitivity to cortisol even in old age.

Next, we studied gene expression in hippocampal samples to determine if the tissue cortisol pathway is affected during aging. While the mRNA expression of 11β-hydroxysteroid dehydrogenase type 2 (*HSD11B2*), the enzyme that catalyzes the conversion of cortisol into cortisone, was not detectable; the expression of 11β-hydroxysteroid dehydrogenase type 1 (*HSD11B1*) was, and moreover, it was significantly (*p* < 0.05) enhanced in the old animals (Figure 1A). No age-related changes in hippocampal expression of two cortisol receptors (*NR3C1* and *NR3C2*) was detected (Figures 1B,C), suggesting that the neurons in this brain area retain their sensitivity to cortisol, even in old age.

**Figure 2:**
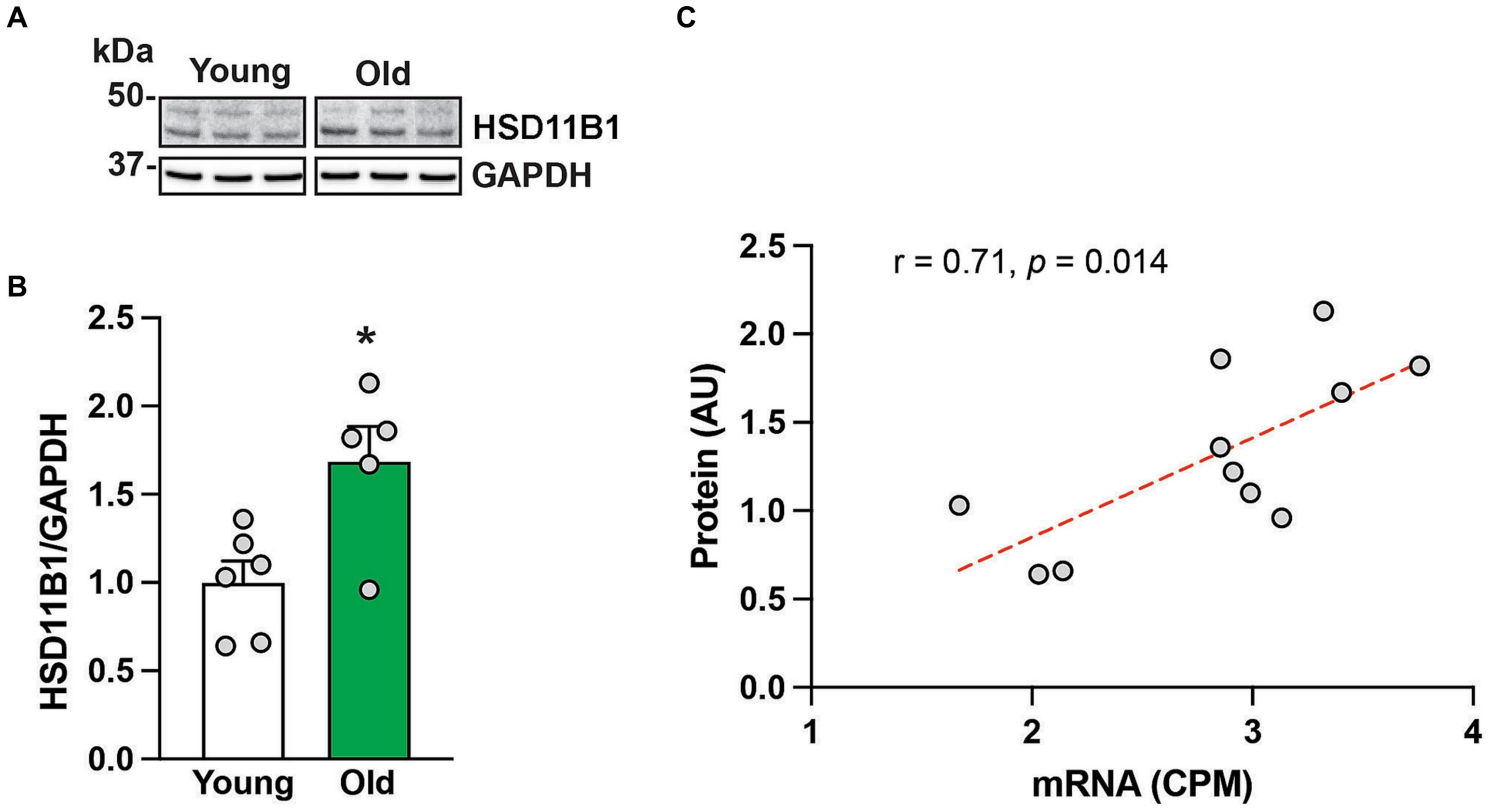
HSD11B protein expression in the hippocampus of 6 young and 5 old male rhesus macaques. Representative western blot images **(A)**, and group means (+SEMs) **(B)**. Correlation between *HSD11B1* gene and HSD11B1 protein expression from the same animals **(C)**. ^*^p < 0.05 (Student’s *t*-test). The results show that HSD11B1 protein levels are higher in older animals and highly correlated with *HSD11B1* mRNA levels, giving further support to the hypothesis that there is enhanced conversion of cortisone to cortisol within the aging hippocampus.

To further understand *HSD11B1* dynamics in the hippocampus of aging animals, we used western blots to examine the age-related differences in HSD11B1 expression at the protein level. Because HSD11B1 protein is highly glycosylated in various tissues (Ozols, 1995; Tomlinson et al., 2004; Chapman et al., 2013; Gathercole et al., 2013), we validated our antibody in samples treated with de-glycosidases. While the expected molecular weight (MW) of HSD11B1 is 36 kDa, both kidney and hippocampal samples show several high molecular weight bands that either disappear or are reduced after de-glycosylation (Supplementary Figure S1). Next, we used the predominant high molecular weight bands to determine HSD11B1 protein levels in the hippocampus of young and old animals (Figure 2A). Hippocampal HSD11B1 protein levels were significantly (*p* < 0.05) elevated in old compared to young animals (Figure 2B; Supplementary Figure S2), and showed a significant (*p* < 0.05) positive correlation between mRNA and protein levels (Figure 2C). In summary, the results indicate that during normative aging there is not only increased overall circulating cortisol but also an imbalance in hippocampal cortisol clearance favoring increased tissue cortisol accumulation.

## 4 Discussion

Many hormones show a circadian pattern in the circulation, which helps to synchronize physiological functions and behaviors with the environment. In male rhesus macaques some of these hormones, including testosterone, dehydroepiandrosterone sulfate (DHEAS) and melatonin, show a significant age-related attenuation of their levels, which may represent a reduction in circadian co-ordination within the body and contribute to age-related disorders such as perturbed sleep–wake cycles (Downs et al., 2008; Urbanski and Sorwell, 2012; Aghazadeh-Sanai et al., 2017). To what extent this attenuation of circadian hormone levels is due to decreased secretion from endocrine organs and/or an increase in their degradation is unclear. However, it is also plausible that the age-related increase in body weight and blood volume that typically occurs in male, but not female, rhesus macaques also contributes to dilution of these hormones in the circulation. It is therefore surprising that cortisol, which also shows a robust circadian pattern in the circulation (Backlund et al., 2017), does not *decrease* with aging in males but instead *increases* significantly (Downs et al., 2008; Urbanski and Sorwell, 2012; Urbanski et al., 2014), and can become neurotoxic and impair memory (Lupien et al., 1998; Hibberd et al., 2000; McEwen, 2002; Herbert et al., 2006; Holmes et al., 2010; McEwen et al., 2015).

The cortisol data from the present study are in agreement with previous reports and emphasize a significant age-related increase in the nadir or baseline serum concentrations of cortisol (Downs et al., 2008; Urbanski and Sorwell, 2012). Because cortisol plays a key role in synchronizing peripheral circadian clocks, as well as influencing physiological functions, such as metabolism, immune response and behavior, chronic elevation of baseline cortisol levels could contribute to the etiology of a wide range of age-related pathologies.

Cortisol exerts its stimulatory actions through both glucocorticoid and mineralocorticoid receptors, and so it’s possible the cortisol-induced brain pathology stems not only from increased circulating cortisol levels but also from an increase in the number of the receptors. However, our RNA-seq results do not support this possibility. Neither the gene that encodes the lower affinity glucocorticoid receptor (*NR3C1*) nor the gene that encodes the higher affinity mineralocorticoid receptor (*NR3C2*) showed an age-related change in expression in the hippocampus. On the other hand, the RNA-seq results did show a significant age-related increase in *HSD11B1* expression, which was also corroborated at the protein level. This enzyme plays a key role in converting cortisone, an inactive glucocorticoid, to cortisol. Therefore, increased HSD11B1 levels during aging would contribute to enhanced availability of cortisol within the hippocampus and thereby potentiate its neurotoxic potential. Importantly, *HSD11B2*, which encodes an enzyme that predominantly converts cortisol to cortisone was undetectable in the hippocampus, emphasizing that HSD11B1 plays the primary role in enzymatically modulating the availability of cortisol in this cognitive brain area.

There is mounting evidence for a pathological role of glucocorticoids and HSD11B1 in obesity and metabolic syndrome. Specifically, obesity is associated with increased HSD11B1 activity in subcutaneous adipose tissue resulting in increased cortisone to cortisol conversion (Stomby et al., 2014). This enzymatic conversion of cortisone to cortisol may therefore serve as an intracellular amplifier of local glucocorticoid activity and play a key role in finely regulating glucocorticoid levels in a tissue specific manner (Staab and Maser, 2010). Such findings have prompted research aimed at inhibiting HSD11B1 as a possible pharmacological target for the prevention of adverse metabolic profiles associated with glucocorticoid excess, such as Cushing syndrome, metabolic syndrome and obesity (Pereira et al., 2012; Morgan et al., 2014). Results from the current study suggest that an age-related increase in HSD11B1 expression may fulfill a similar regulatory role within the brain, and that pharmacological targeting of this enzyme may have therapeutic potential in reducing local deleterious effects associated with excessive cortisol levels.

In summary, the results from the present study are the first ones to demonstrate an age-related increase in hippocampal expression of *HSD11B1* in a translational nonhuman primate species. Importantly, because of the many physiological, neuronal and endocrine similarities between rhesus macaques and humans (Messaoudi et al., 2011; Sorwell et al., 2012; Stonebarger et al., 2021), the results support the idea that an age-related increase in *HSD11B1* expression in the hippocampus, together with elevated circulating cortisol levels, predispose this vulnerable brain area to neurotoxicity, cognitive decline and ultimately to development of dementia in the elderly. The studies also lay a foundation for pharmacological interventions that target HSD11B1, as a possible therapeutic for these age-related disorders (Sandeep et al., 2004; MacLullich et al., 2012; Sooy et al., 2015).

## Data availability statement

The original contributions presented in the study are publicly available. This data can be found at: https://www.ncbi.nlm.nih.gov/geo/, GSE249907.

## Ethics statement

The animal study was approved by ONPRC Institutional Animal Care and Use Committee. The study was conducted in accordance with the local legislation and institutional requirements.

## Author contributions

AL: Methodology, Validation, Writing – original draft. SL: Methodology, Writing – review & editing. RC-J: Methodology, Writing – review & editing. M-LA: Writing – review & editing. SGK: Resources, Writing – review & editing. HFU: Conceptualization, Funding acquisition, Writing – original draft, Writing – review & editing.
