# Supplementary material for: Age-related increase in the expression of 11β-hydroxysteroid dehydrogenase type 1 in the hippocampus of male rhesus macaques
Source: Front Aging Neurosci. 2024 Mar 15;16:1328543. doi: 10.3389/fnagi.2024.1328543 (PMC10978655; doi:10.3389/fnagi.2024.1328543)
Supplement: Supplementary file 1 [file Data_Sheet_1.PDF]

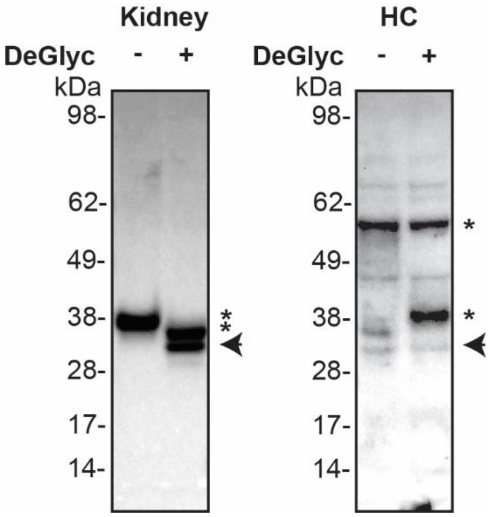

Supplementary Fig.1 Western blot for HSD11B1 in kidney and HC samples to test whether the different molecular weights of HSD11B1 are due to glycosylation. De-glycosylation experiment was performed using the glycoprotein de-glycosylation kit from Millipore Sigma. Briefly, 200-μg protein extracts were incubated with reaction buffer and 1 μl each of N-glycosidase F, α2-3,6,8,9-neuraminidase, endo-α-N-acetylgalactosaminidase, β1,4-galactosidase and 1 μl β-N-acetylglucosaminidase and incubated for 3 h at 37°C, indicated with (+). Arrowheads indicate fully de-glycosylated form of HSD11B1 (Approx. 35 kDa); asterisks indicate glycosylated forms

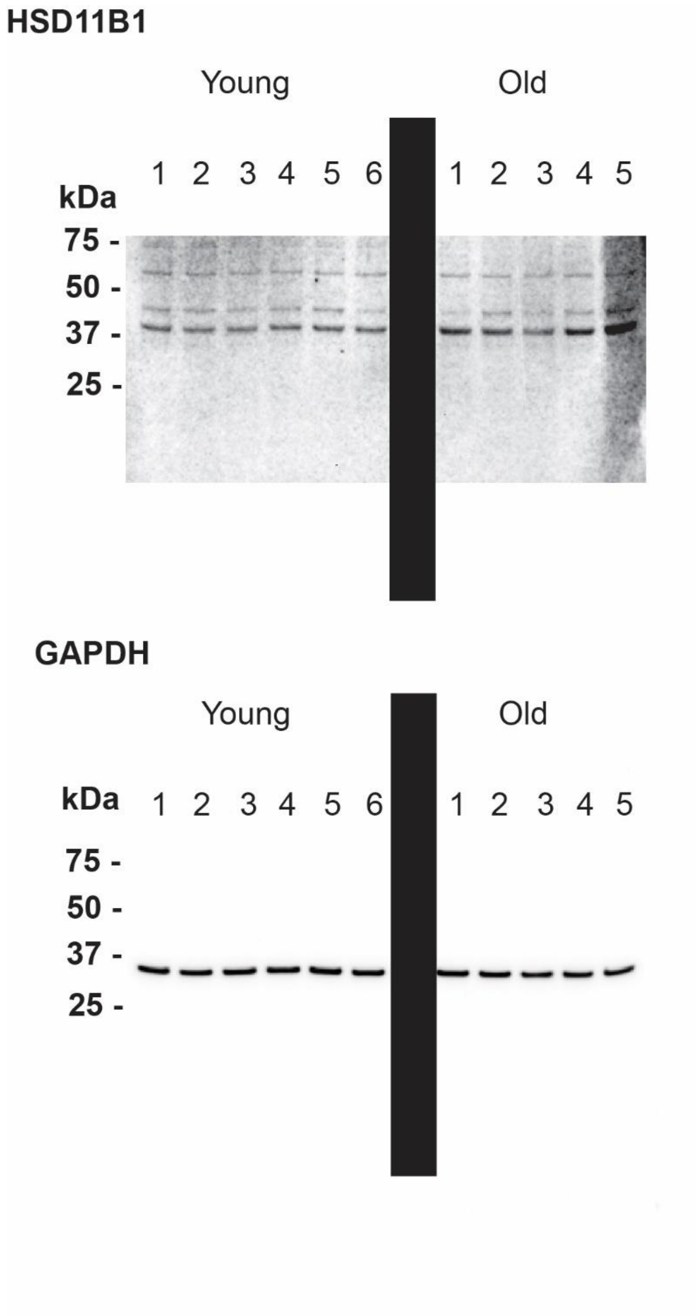

Supplementary Fig.2 Western blot for HSD11B1 and GAPDH (a housekeeping protein) in all of the hippocampal samples from young and old animals
